# Neuronal Sigma-1 Receptors: Signaling Functions and Protective Roles in Neurodegenerative Diseases

**DOI:** 10.3389/fnins.2019.00862

**Published:** 2019-08-28

**Authors:** Daniel A. Ryskamp, Svetlana Korban, Vladimir Zhemkov, Nina Kraskovskaya, Ilya Bezprozvanny

**Affiliations:** ^1^Department of Physiology, UT Southwestern Medical Center at Dallas, Dallas, TX, United States; ^2^Laboratory of Molecular Neurodegeneration, Peter the Great St. Petersburg State Polytechnic University, Saint Petersburg, Russia

**Keywords:** synapse, calcium, neuroprotection, Alzheimer’s, Huntington and Parkinson diseases, ALS (amyotrophic lateral sclerosis)

## Abstract

Sigma-1 receptor (S1R) is a multi-functional, ligand-operated protein situated in endoplasmic reticulum (ER) membranes and changes in its function and/or expression have been associated with various neurological disorders including amyotrophic lateral sclerosis/frontotemporal dementia, Alzheimer’s (AD) and Huntington’s diseases (HD). S1R agonists are broadly neuroprotective and this is achieved through a diversity of S1R-mediated signaling functions that are generally pro-survival and anti-apoptotic; yet, relatively little is known regarding the exact mechanisms of receptor functioning at the molecular level. This review summarizes therapeutically relevant mechanisms by which S1R modulates neurophysiology and implements neuroprotective functions in neurodegenerative diseases. These mechanisms are diverse due to the fact that S1R can bind to and modulate a large range of client proteins, including many ion channels in both ER and plasma membranes. We summarize the effect of S1R on its interaction partners and consider some of the cell type- and disease-specific aspects of these actions. Besides direct protein interactions in the endoplasmic reticulum, S1R is likely to function at the cellular/interorganellar level by altering the activity of several plasmalemmal ion channels through control of trafficking, which may help to reduce excitotoxicity. Moreover, S1R is situated in lipid rafts where it binds cholesterol and regulates lipid and protein trafficking and calcium flux at the mitochondrial-associated membrane (MAM) domain. This may have important implications for MAM stability and function in neurodegenerative diseases as well as cellular bioenergetics. We also summarize the structural and biochemical features of S1R proposed to underlie its activity. In conclusion, S1R is incredibly versatile in its ability to foster neuronal homeostasis in the context of several neurodegenerative disorders.

## Introduction

Sigma-1 receptor (S1R) is a ligand-operated protein that modulates activity of several client proteins from its position within the membrane of the endoplasmic reticulum (ER). It is widely expressed in multiple organs including the nervous system (Gundlach et al., 1986) and it has important roles in modulation of neuronal physiology (Maurice et al., 2006a) and synaptic plasticity (Takebayashi et al., 2004; Tsai et al., 2009; Kourrich et al., 2012). Autosomal recessive loss-of-function mutations in S1R are primarily associated with amyotrophic lateral sclerosis/frontotemporal dementia (ALS/FTD) (Luty et al., 2010; Al-Saif et al., 2011; Kim et al., 2014; Li et al., 2015; Ullah et al., 2015; Gregianin et al., 2016; Horga et al., 2016; Watanabe et al., 2016), but polymorphisms in S1R also affect risk of developing Alzheimer’s disease (AD) (Uchida et al., 2005; Maruszak et al., 2007; Huang et al., 2011; Fehér et al., 2012). Many S1R agonists are neuroprotective and loss of S1R accelerates neurodegenerative phenotypes (Maurice and Lockhart, 1997; Nakazawa et al., 1998; Smith et al., 2008; Mavlyutov et al., 2011, 2013; Mancuso et al., 2012; Ono et al., 2014; Ryskamp et al., 2017, 2019; Maurice et al., 2018). Neuroprotection from S1R activation is achieved by a diversity of signaling functions that promote cellular homeostasis and synaptic stability. In this review we summarize therapeutically relevant mechanisms by which the ligand-operated chaperone S1R modulates neurophysiology, counteracting its dysregulation from pathogenic stressors.

## Modulation of Neurophysiology by S1R

Sigma-1 receptor is a 223 amino acid-long transmembrane protein residing in the ER membrane. S1R preferentially localizes to the specific microdomains of the ER called mitochondrial-associated membranes (MAM), where it can regulate InsP_3_R-dependent calcium flux from the ER to mitochondria (Hayashi and Su, 2007), lipid dynamics (Hayashi and Su, 2005), MAM stability (Watanabe et al., 2016), and the ER stress response (Mori et al., 2013). The MAM domain is also important for synthesis and transport of lipids and protein folding (Weng et al., 2017b). Under resting conditions, S1R forms an inert complex with GRP78/BiP protein (Hayashi and Su, 2007). When activated by agonists or ER calcium depletion, S1R dissociates from BiP and redistributes to the entire ER network (Hayashi and Su, 2007), freeing it to interact with and modulate several client proteins including InsP_3_Rs inside and outside of the MAM domain as well as plasmalemmal ion channels, GPCRs, and kinases (summarized in Table 1).

**TABLE 1:** S1R binding partners and biological outcomes mediated by these interactions.

**S1R-interactng protein**	**Method(s) revealing interaction**	**Regulation by S1R expression level**	**Regulation by S1R activation**	**References**
**Plasmalemmal ion channels**				
Kv1.2	Co-IP	Increased surface expression	Agonists (cocaine) enhanced association between S1R and Kv1.2; increased surface expression	Kourrich et al., 2013; Delint-Ramirez et al., 2018
Kv1.3	Co-IP	Co-expression increased Kv1.3 inactivation	Inhibition by SKF-10047	Kinoshita et al., 2012
Kv1.4	Co-IP	Overexpression of S1R dose-dependently increased Kv1.4 inactivation	SKF-10047 reduced Kv1.4 outward currents	Aydar et al., 2002
Kv1.5		Overexpression of S1R inhibited Kv1.5 currents	SKF-10047 reduced Kv1.5 outward currents	Maurice et al., 2006a
Kv2.1	Imaging			Mavlyutov et al., 2010
L-type Ca^2+^ channels	Co-IP		Inhibition by S1R agonists (SKF-10047)	Church and Fletcher, 1995; Zhang and Cuevas, 2002; Tchedre et al., 2008; Mueller et al., 2013
N-type Ca channels	Co-IP	Inhibition by S1R overexpression	Inhibition by SKF-10047, PRE-084	Zhang and Cuevas, 2002; Johannessen et al., 2009; Gao et al., 2012; Zhang et al., 2017
Nav1.5	Co-IP, pulldown, AFM	Knockdown decreased Nav1.5 currents	Agonists (PTZ) promoted dissociation of Nav1.5	Balasuriya et al., 2012
ASIC1a	Co-IP, pulldown, AFM		S1R activation decreased ASIC1a currents	Herrera et al., 2008; Carnally et al., 2010
hERG	Co-IP, AFM	Potentiation by S1R overexpression	Ligands depressed hERG currents	Crottes et al., 2011; Balasuriya et al., 2014
STIM1/Orai1	Co-IP, imaging, AFM	Overexpression inhibited SOC; S1R KD enhanced SOC	Agonists inhibited SOC while antagonists enhanced SOC	Srivats et al., 2016
**ER channels**				
InsP_3_R1	Calcium imaging		Agonists suppressed ER calcium release mediated by InsP_3_R1	Ryskamp et al., 2017
InsP_3_R3	Co-IP	Stabilization by S1R, overexpression increased IP3-induced Ca-release	Activation of IP3-induced Ca-release by agonists	Hayashi and Su, 2007; Wu and Bowen, 2008; Delint-Ramirez et al., 2018
**ER resident proteins**				
BiP/GRP78	Co-IP, pull-down, NMR	Stable complex formation with BiP	Agonists dissociated S1R from BiP	Hayashi and Su, 2007; Ortega-Roldan et al., 2013
Insig1; UDP-galactose:Ceramide Galactosyltransferase (CGalT)	Co-IP	Overexpression of S1R increased degradation of CGalT; S1R KO increased protein levels of CGalT	Agonist (PTZ) increased association of S1R with Insig1	Hayashi et al., 2012
IRE1a	Co-IP, proximity biotinylation labeling	S1R overexpression sustained IRE1 phosphorylation and signaling (Mori et al., 2013), S1R KO increased IRE1 activity (Rosen et al., 2019)	Fluvoxamine led to anti-inflammatory response	Hayashi and Su, 2007; Mori et al., 2013; Alam et al., 2017; Rosen et al., 2019
Ankyrin	Co-IP	S1R forms stable ternary complex with ankyrin and IP_3_R3s	Agonists dissociated ankyrin from S1R and potentiated IP_3_-induced Ca-release	Hayashi and Su, 2001
**PM receptors/proteins**				
CB1R	Bimolecular fluorescence complementation assay	S1R regulates formation of a CB1-HINT1-GluN1 complex	S1R opposed CB1R-mediated suppression of NMDAR activity	Sanchez-Blazquez et al., 2014
D1R	BRET	Formation of D1R-S1R heteromers	S1R agonists enhanced D1R signaling	Navarro et al., 2010
D2R	Co-IP, BRET	Formation of D2R-S1R heteromers	S1R agonist (cocaine) inhibited D2R signaling	Navarro et al., 2013
MOR	Co-IP, [35S]GTPγS binding	Potentiation by S1R knockdown	Potentiation by S1R antagonist	Kim et al., 2010
Integrin b1	Co-IP	n/d	Agonist (SKF-10047) reduced interaction and reduced cell adhesion	Palmer et al., 2007
BDNF		Knockdown suppressed secretion of mature BDNF	Agonists promote secretion of mature BDNF	Fujimoto et al., 2012
TrkB	Co-IP		Activation of S1R promoted TrkB signaling	Kimura et al., 2013; Ka et al., 2016
PDGFR	Pull-down, co-IP, FRET			Yao et al., 2011
Dopamine transporter (DAT)	Co-IP, BRET, functional assays		Agonists modulated stimulant binding to DAT and stimulant-evoked DA efflux via DAT and calcium signals	Hong W.C. et al., 2017; Sambo et al., 2017
**Mitochondrial proteins**				
VDAC2; StAR	Co-IP	Reduction of cholesterol efflux under S1R KD conditions		Marriott et al., 2012
**Proteins in the Cytosol**				
Rac1	Co-IP	Interacts as part of multiprotein complex involving S1R, IP3R, Rac, BiP	Agonist (PTZ) increased association	Natsvlishvili et al., 2015
ELMOD 1-2	Co-IP	Binding inhibited GAP activity		Ivanova et al., 2014
**Other**				
Emerin	Co-IP, native gel electrophoresis	Association with HDAC1/2, BAF, Emerin	Increased association with HDAC1/2, BAF, emerin after cocaine treatment (*in vivo*)	Tsai et al., 2015a
Androgen receptor (AR)	Co-IP	Increased AR degradation under S1R KD conditions	S1R inhibitors prevented nuclear transclocation and increased degradation of AR	Thomas et al., 2017

Sigma-1 receptor agonists do not noticeably alter ER calcium signaling under resting conditions (Hayashi et al., 2000), but they can influence ER calcium release triggered by Gq-coupled receptors (Hayashi and Su, 2007; Ryskamp et al., 2017). S1R chaperones InsP_3_R3 to the MAM domain and prevents its degradation, enhancing Ca^2+^ flux into mitochondria (Hayashi and Su, 2007). This augments ATP production (Griffiths, 2009), but, in excess, it could also trigger the mitochondrial permeability transition (Lemasters et al., 2009). By contrast, InsP_3_R1, which is the predominant InsP_3_R isoform in neurons and has important signaling functions outside of the MAM, is negatively regulated by agonist-stimulated S1R in certain cell types like striatal medium spiny neurons (MSNs) (Ryskamp et al., 2017). Regulation ER calcium homeostasis and signaling by S1R has important implications for neurodegenerative diseases and this will be discussed later.

Engagement of S1R with plasmalemmal channels and receptors is responsible for S1R-dependent fine-tuning of neuronal excitability (Kourrich et al., 2012). As many of S1R’s interaction partners function in the plasma membrane, it was proposed that activated S1R translocates from the ER to the plasma membrane where it binds to client proteins and acts as a chaperone or an auxiliary subunit (McCann and Su, 1990; Morin-Surun et al., 1999; Hayashi et al., 2000; Aydar et al., 2002). However, this assumption is often based on experiments in which S1R and/or its interaction partner are overexpressed, raising several caveats that call this interpretation into question (Su et al., 2016). Alternatively, S1R may interact with plasma membrane proteins from its position in the ER like STIM proteins (Mavlyutov et al., 2010, 2015a) and/or regulate the maturation and/or trafficking of certain proteins to the plasma membrane (Crottes et al., 2011; Delint-Ramirez et al., 2018).

Sigma-1 receptor activation alters neuronal excitability through its interactions with voltage-gated ion channels. Voltage-gated sodium (Nav) channels augment neuronal depolarization and mediate action potentials. S1R ligands dissociate S1R from Nav1.5, leading to suppressed Nav1.5 activity (Johannessen et al., 2009; Balasuriya et al., 2012). This action can be evoked by the endogenous S1R agonist *N*,*N*-dimethyltryptamine (DMT) and is partially opposed by the endogenous S1R antagonist progesterone (Johannessen et al., 2011). S1R agonists also limit excitability by inhibiting other Nav channels including Nav1.2 and Nav1.4 (Johannessen et al., 2009; Gao et al., 2012). Voltage-gated potassium (Kv) channels respond to membrane depolarization during action potentials by releasing positively charged potassium ions from the cytosol to restore a hyperpolarized resting membrane potential and limit hyperexcitability. When S1R is activated by cocaine, S1R binds to the voltage-gated potassium channel Kv1.2 and enhances trafficking of Kv1.2 to the plasma membrane, decreasing excitability of dopamine D1 receptor (D1R)-expressing MSNs in the shell of the nucleus accumbens (Delint-Ramirez et al., 2018). S1R expression and activity also regulates the cardiac Kv channel hERG through control of maturation and trafficking (Crottes et al., 2011) and this function appears to be dependent on cholesterol and not S1R ligands, possibly suggesting a role for lipid rafts in S1R client protein assembly and trafficking (Balasuriya et al., 2014). S1R appears to basally regulate Kv1.3 and Kv1.4 independent of agonist-stimulation (Aydar et al., 2002; Kinoshita et al., 2012). By means of such interactions, S1R regulates neuronal excitability.

Sigma-1 receptor also influences synaptic functions through modulation of the NMDA receptor (NMDAR) activity. Physiological NMDAR activation can induce hippocampal long-term potentiation (LTP) (Lu et al., 2001), spine maturation (Tada and Sheng, 2006) and learning (Morris et al., 1986), but pathophysiological levels of NMDAR activity triggers excitotoxicity (Rothman and Olney, 1987). S1R facilitates NMDA receptor signaling and neurotransmission in hippocampal neurons (Monnet et al., 1990, 1992, 1995), possibly through altering responses to calcium signals (e.g., inhibiting Ca^2+^-activated SK channels) and promoting expression of NMDA receptor subunits and their trafficking to the plasma membrane (Martina et al., 2007; Pabba et al., 2014). S1R can also obviate negative-regulation of NDMARs by cannabinoid 1 receptor (CB1R) (Sanchez-Blazquez et al., 2014). These interactions enhance neuronal firing and maturation of mushroom spines from NMDA receptor activation (Monnet et al., 1990; Martina et al., 2007; Pabba et al., 2014). Modulation of calcium signaling by S1R may regulate synaptic plasticity through stimulation of CaMKII, PKC, and ERK (Moriguchi et al., 2011).

Sigma-1 receptor agonists may promote synaptic plasticity and neuronal resilience in large part through their common ability to upregulate BDNF secretion and TrkB receptor signaling both *in vitro* and *in vivo* (Kikuchi-Utsumi and Nakaki, 2008; Peviani et al., 2014). For example, pridopidine, a potent S1R receptor agonist, promotes neurotrophic signaling via BDNF, ERK, and AKT pathways (Ono et al., 2014; Geva et al., 2016; Kusko et al., 2018; Ionescu et al., 2019). S1R agonists appear to activate TrkB both through BDNF-dependent (Kimura et al., 2013) and independent mechanisms (Ka et al., 2016). This may involve regulation of BDNF expression and processing as well as direct interactions of S1R with the TrkB receptor (Fujimoto et al., 2012; Kimura et al., 2013; Ka et al., 2016). S1R also stimulates signaling by other receptor tyrosine kinases including the epidermal growth factor receptor (EGFR) (Takebayashi et al., 2004) and the platelet-derived growth factor receptor (PDGFR) (Yao et al., 2011). Stimulation of neurotrophic receptors confers neuroprotection through control of gene expression.

Indirect regulation of transcriptional activity by S1R contributes to its neuroprotective properties. For example, S1R may prevent neuronal death by upregulating expression of the antiapoptotic mitochondrial protein Bcl-2 (Meunier and Hayashi, 2010; Zhang et al., 2012). S1R regulates transcription through interactions with inositol-requiring enzyme 1 (IRE1) and emerin. S1R facilitates dimerization of the ER stress sensor and endonuclease IRE1 at the MAM domain, leading to splicing-dependent activation of the transcription factor XBP1, which goes on to upregulate several ER chaperones (Mori et al., 2013). S1R also decreases IRE1-driven inflammation (Rosen et al., 2019), which may be important for microglial reactivity and migration to and from injury sites (Moritz et al., 2015). As the ER membrane is contiguous with the nuclear envelope, activated S1R can move to the nuclear envelope where it regulates transcription through its recruitment of emerin and then chromatin-remodeling factors (Tsai et al., 2015a).

A microarray study involving knockdown of S1R in cultured hippocampal neurons revealed altered transcription in pathways controlling protein ubiquitination, sterol biosynthesis, oxidative stress, and actin dynamics (Tsai et al., 2012). Knockdown of S1R reduces the size of dendritic spine size in hippocampal neurons, indicating that it actively supports stability of mature spines (Tsai et al., 2009; Fisher et al., 2016; Ryskamp et al., 2019). This was initially proposed to involve its role in regulating oxidative stress and Rac-GTP signaling (Tsai et al., 2009), but may also involve modulation of calcium homeostasis in conditions of disease (Ryskamp et al., 2019). Knockout of S1R is associated with increased formation of reactive oxygen species (ROS) and decreased expression and activity of NRF2, which promotes expression and activation of antioxidant molecules under conditions of stress (Wang et al., 2015). This may explain how S1R suppresses generation of ROS (Meunier and Hayashi, 2010). Interestingly, spine shrinkage from knocking down S1R was prevented by reducing oxidative stress (Tsai et al., 2009).

Finally, in addition to protein–protein modulation, S1R was shown to interact with lipids. *S*1R localizes to lipid rafts – detergent-resistant microdomains of the ER – where it binds cholesterol at sterol-binding sites and S1R agonists such as SKF-10047 displace S1R and its binding partners from lipid rafts possibly through out-competing cholesterol binding (Hayashi and Su, 2003; Palmer et al., 2007). S1R targets to galactosyl-rich microdomains of the ER and is potentially involved in regulation of the differentiation of oligodendocytes and myelination (Hayashi and Su, 2004) as well as lipid transport to the myelin membrane (Weng et al., 2017a). S1R also supports axonal growth through promoting myristoylation of p35, which increases its degradation and thereby decreases p25/CDK5-dependent hyperphosphorylation of Tau (Tsai et al., 2015b).

**FIGURE 1:**
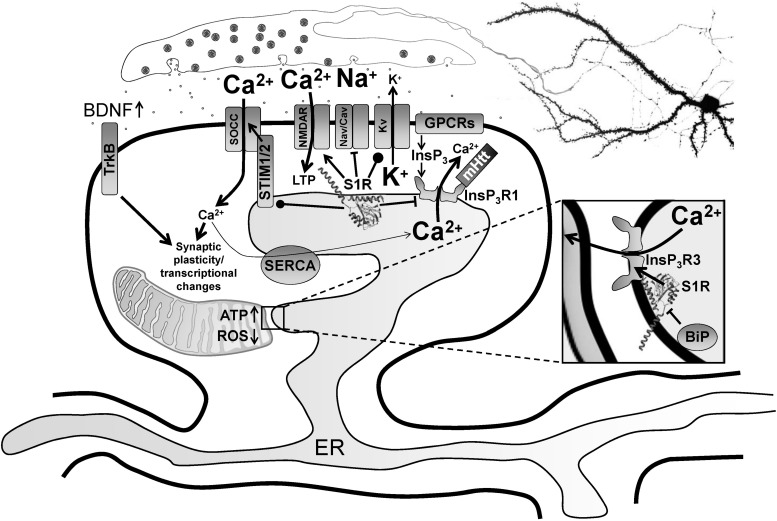
Modulation of neurophysiology by S1R. Normally residing at the MAM, S1R is released from BiP upon activation from ER calcium depletion, ER stress or agonist stimulation, freeing it to interact with its client proteins. Within the MAM, S1R regulates lipid dynamics and chaperones InsP_3_R3 to the MAM, facilitating calcium flux from the ER to mitochondria. This enhances ATP production. S1R’s actions on transcriptional pathways counteract oxidative stress through upregulation of antioxidants. Once activated, S1R redistributes to the entire ER network where it interacts with additional targets including InsP_3_R1, STIM1 and several plasma membrane ion channels and receptors. For example, S1R activation by pridopidine in striatal MSNs attenuates ER calcium release from InsP_3_R1 when it is hyperactive in HD from mutant Huntingtin protein, leading to suppression of synaptotoxic signals mediated by store-operated calcium entry channels (SOCCs). Conversely, nSOC pathway activity is important for mushroom spine stability in AD, but it is downregulated from reduced ER calcium leakage in AD models. In hippocampal neurons, S1R decreases ER calcium levels, possibly though positive regulation of presenilin leak channels (not shown). This restores nSOC pathway activity and promotes mushroom spine stability. S1R activation also limits excitotoxicity by decreasing activity of Nav and Cav channels, while promoting activity of some Kv channels. S1R enhances NMDAR activity, which is important for induction of LTP as well as activation of calcium-dependent transcription factors. S1R also modulates several GPCRs, which can influence several physiological processes including monoamine neurotransmission. Moreover, S1R activation promotes synaptic plasticity and neuronal survival through upregulation of BDNF expression and secretion as well as direct stimulation of TrkB receptors. S1R monomers are shown with the crystal structure adapted from Schmidt et al. (2016), but S1R ligand-dependent oligomerization/monomerization may confer specificity in its diverse actions.

This review only scratches the surface with regard to S1R’s multiplicitous roles in neurophysiology/neuroprotection and provides a glimpse into the specificity of its actions in differing cell types. It is tempting to speculate that the nature of modulation by S1R depends on the levels of S1R and its interaction partners in a given cell type (e.g., preferential interactions of S1R with cell-type abundant effectors) as well as many circumstantial factors such as the current physiological/pathophysiological state of the cell and signaling events (e.g., presence/absence of S1R ligands). To better understand possible actions of S1R, we summarized S1R interaction partners and the effects of S1R ligands and knockdown/overexpression on these protein–protein interactions (Figure 1 and Table 1).

## Molecular Analysis of S1R

Sigma-1 receptor is a promising therapeutic target in the treatment of neurodegenerative diseases as it stabilizes the function of several intracellular systems through its role as a chaperone when activated by a variety of ligands with neuroprotective properties. Despite S1R pluripotency, relatively little is known regarding the mechanisms of receptor functioning and regulation at the molecular level. There is evidence that the structural organization of S1R and its conformational state are important determinants of S1R activity. However, the structural basis for S1R functionality remains poorly understood.

Originally, S1R was characterized as a type 1 transmembrane protein with only one transmembrane domain (Hanner et al., 1996). Hydrophobicity analysis confirmed a single-pass transmembrane topology of S1R (Kekuda et al., 1996), however, subsequent studies predicted a two transmembrane domain model of S1R topology (Aydar et al., 2002; Ortega-Roldan et al., 2013). For example, Aydar and colleagues proposed two-transmembrane domains (TM1 a.a. 11–29 and TM2 a.a. 80–100) based on antibody staining experiments with expression of S1R fused to GFP at the N- or C-terminus in *Xenopus* oocytes (Aydar et al., 2002). Immunolabeling of the GFP-tags was absent without membrane permeabilization, but was detected after permeabilization with 0.5% acetone, leading them to conclude that both the N- and the C-termini are located near the plasma membrane but within the cytoplasm. By contrast, the topological model proposed by Hayashi and Su situates S1R in ER membranes with both N- and C-terminal regions oriented to the ER lumen (Hayashi and Su, 2007). This was based on immunocytochemical staining of endogenous S1R in CHO cells with antibodies targeting N- and C-termini. Similar to results of Aydar et al. (2002), no labeling was detected without permeabilization, suggesting that S1R is not in the plasma membrane. Permeabilization of plasma and ER membranes with CHAPS or Triton X-100 enabled staining for all antibodies with a distribution similar to the shape of the ER. When the plasma membrane was permeabilized with streptolysin-O, staining was present only for the antibody targeting the loop domain. The discrepancy between the topology models of Aydar et al. (2002) and Hayashi and Su (2007) may have arisen from altered membrane insertion of GFP-fused S1R and/or cell type specific differences in S1R localization. For example, Hayashi and Su note that fusion of YFP to the C-terminus of S1R, but not the N-terminus, mirrors the distribution of endogenous S1R (Hayashi and Su, 2007).

The two-pass transmembrane model was widely accepted for a long time and has been used as a structural basis for molecular modeling and ligand docking studies (Laurini et al., 2011). However, the crystal structure for human S1R was recently solved revealing a single transmembrane domain structure (Schmidt et al., 2016). According to this study, a short N-terminus faces the ER lumen while the C-terminal domain of protein is oriented to the cytosolic side (Schmidt et al., 2016).

Adding another possible model of S1R topology to the mix, Mavylutov et al. (2018) fused ascorbate peroxidase 2 (APEX2) to the N- or C-terminus of S1R and used electron microscopy to visualize deposition of 3,3′-diaminobenzidine outside or inside of the ER of ND7/23 cells and dorsal root ganglion (DRG) neurons. This experiment indicated that the N-terminus of S1R faces the cytosol, whereas the C-terminus is located in the ER lumen. This is consistent with the one transmembrane model suggested by the crystal structure, but suggests the orientation of S1R positions the bulk of its structure in the lumen of the ER with only a short N-terminus facing the cytosol. All of the experiments probing the topology and orientation of S1R have caveats that are important to keep in mind including specificity of antibodies and membrane permeabilization as well as alterations to S1R from protein fusions and crystallization conditions.

**FIGURE 2:**
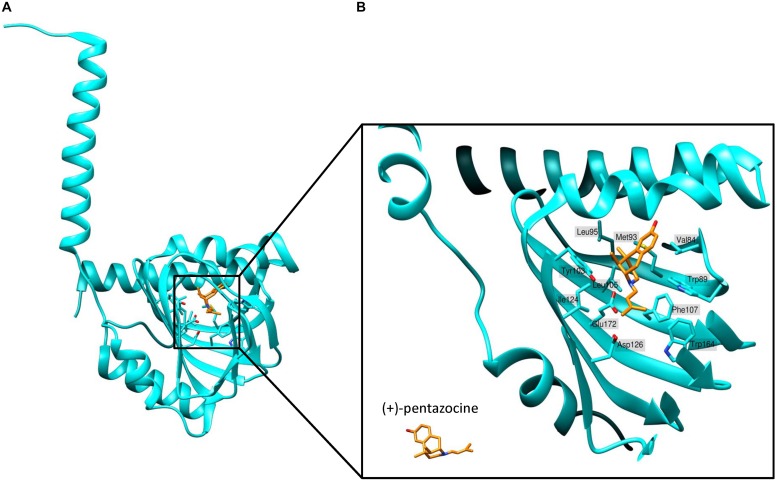
Structure of the ligand-binding site of S1R bound to agonist (+)-pentazocine. **(A)** The overall structure of a sigma-1 receptor subunit bound to (+)-pentazocine (PDB ID: 6DK1) based on (Schmidt et al., 2018). **(B)** A close up of the binding pocket showing the key amino acids involved in coordination of the ligand. (+)-pentazocine is shown in orange. Glu172 interacts with (+)-pentazocine’s nitrogen atom (blue) and both Tyr103 and Asp126 facilitate this through creating hydrogen bonds with Glu172. Other amino acids including Val84, Trp89, Met93, Leu95, Tyr103, Leu105, Phe107, Ile124, and Trp164 help to form the primarily hydrophobic binding pocket and stabilize the ligand through Van der Waals interactions.

Schmidt et al. (2016) determined the first crystal structure of full-length human S1R using X-ray analysis. They expressed FLAG-tagged S1R in Sf9 insect cells, purified it using the detergent lauryl maltose neopentyl glycol (LMNG) and crystallized it using the lipidic cubic phase (LCP) method (Caffrey et al., 2012). According to the crystal structure, S1R homomers consist of three protein subunits, with each protomer having one transmembrane domain (Figure 2A). The transmembrane alpha-helices of trimers (encompassing amino acid residues 8 to 32) are separated from each other and located in the corners of the complex, while the C-terminal cytosolic domains of each protomer organize the trimeric structure and form highly conserved ligand-binding sites. The membrane proximal surface of each C-terminal domain is tightly associated with the cytosolic surface of the ER membrane. Each C-terminal domain contains a cupin-like β-barrel that can envelop a ligand (Figure 2A).

An accurate model of the S1R ligand binding pocket is necessary for rational drug design aimed at the targeted treatment of neurodegenerative diseases. Mapping of the S1R ligand binding site was carried out in a large number of studies using mutational analysis and photoreactive probe labeling (Yamamoto et al., 1999; Chen et al., 2007; Pal et al., 2007, 2008). The first identified amino acids that are important for ligand binding include Ser99, Tyr103, Leu105, and Leu106 (Yamamoto et al., 1999). The results of these studies are highly consistent with the structural model of S1R obtained via X-ray crystal analysis.

Sigma-1 receptor crystal structures harboring chemically distinct ligands (the high-affinity, selective S1R ligands PD144418 and 4-IBP) show that both ligands bind in similar positions, forming electrostatic interactions with the highly conserved amino acid residue Glu172 (Figure 2B). The amino acid Asp126 which is also essential for ligand binding forms a hydrogen bond with Glu172 (Schmidt et al., 2016). With the exception of only two amino acid residues, the S1R active site is hydrophobic and is occluded from aqueous solution. Other amino residues involved in ligand coordination are as follows: Val84, Trp89, Met93, Leu95, Tyr103, Leu105, Phe107, Ile124, Trp164, and Leu182 (Figure 2B). Additionally, Tyr103 creates a hydrogen bond with Glu172, which is apparently necessary for the formation of the binding pocket (Figure 2B). Indeed, in earlier works a significant decrease in the binding activity of the mutant Tyr103Phe was shown (Yamamoto et al., 1999). The highly occluded structure of the binding pocket raises the questions about the pathway of ligand entry and explains the very slow ligand binding kinetics.

Schmidt et al. (2018) conducted additional structural studies and molecular dynamics (MD) simulation experiments to reconstruct the ligand binding mechanism in detail. They solved crystal structures of S1R bound to the classical antagonists haloperidol and NE-100 as well as the agonist (+)-pentazocine. The obtained structures were highly similar to each other and did not differ significantly from the previously determined trimeric structures of S1R. They share a similar organization of the ligand-binding pocket. The overall conformations of S1R in complex with the antagonists or agonist remain almost identical with the exception of a difference in the position of (+)-pentazocine in the ligand binding site. On the basis of structural data and MD simulations the authors suggest that agonist binding leads to conformational changes of S1R compared to the unliganded form of receptor or antagonist bound S1R. MD simulations were used to characterize conformational rearrangements occurring during ligand association. Ligand association was characterized as a two-step process: (1) the hydrogen bonds between Trp136 and Ala161 break, leading to receptor “lid” opening, and (2) the interior of the receptor separates, exposing the binding pocket and allowing ligand entry.

Schmidt et al. (2018) provided valuable insights on the structural basis for a ligand binding mechanism and describe the potential conformational differences between agonist and antagonist bound S1R. However, they do not explain the functional role of agonist/antagonist actions and the physiological relevance of agonist-induced structural rearrangements of S1R. The relationship between the ligand-receptor association and the subsequent biological response remains unclear.

**FIGURE 3:**
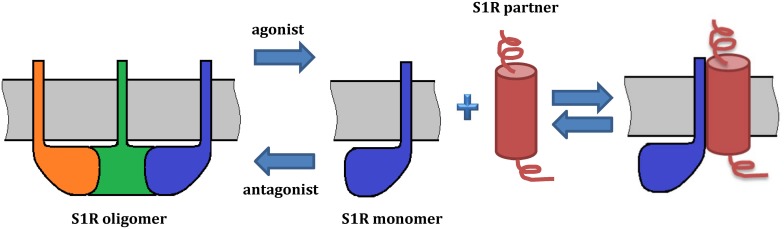
Model of S1R oligomerization and its functional role. The model is based on Mishra et al. (2015) and Hong W.C. et al. (2017). On the left, a S1R trimer is shown. Agonists promote dissociation of S1R into monomers, which may redistribute to other subcellular compartments and associate with client proteins. By contrast, antagonists prevent such interactions by stabilizing S1R oligomerization. Ligands regulate the interactions of S1R with its protein partners. While oligomeric forms of S1R have a demonstrated ability to bind ligands, S1R monomers may lose this property. As several oligomeric forms of S1R have been reported, they may also have functional roles and oligomer-specific interaction partners.

Extensive evidence indicates that S1R exists in multiple oligomeric states (Chu et al., 2013; Gromek et al., 2014; Mishra et al., 2015) and recent studies suggest that ligand binding induces a shift in the oligomeric state of S1R (Gromek et al., 2014; Mishra et al., 2015; Hong W.C. et al., 2017), which could in turn lead to the various functional responses. For example, high-molecular weight forms of S1R have been detected in rat liver microsomal membranes using radioactive photosensitive labels (Pal et al., 2007). Oligomeric forms of S1R corresponding to hexamers, tetramers, octamers were also identified by size-exclusion chromatography (SEC) (Gromek et al., 2014). Analysis by SDS-PAGE after chemical crosslinking of individual oligomeric forms of MBP fused S1R has also confirmed the presence of monomers, tetramers and high molecular weight S1R oligomers (Mavlyutov et al., 2011). Cell-based fluorescence resonance energy transfer (FRET) studies have also confirmed the existence of several oligomeric states (Mishra et al., 2015) and revealed that agonists stabilize low-molecular-weight species, whereas antagonists favor oligomers. This model suggests that monomeric form of S1R is an “active” conformation involved in interactions with client proteins (Figure 3). On the other hand, Gromek et al. (2014) found similar stabilizing effects of agonists and antagonists on S1R oligomeric states. However, the detergents used in the purification procedure of their experiments do not reflect the native lipid environment of membrane proteins, potentially limiting the validity of this finding. While the crystal structure of S1R has a trimeric fold, size-exclusion, cross-linking and multi-angle light scattering (SEC-MALS) experiments have revealed a wide range of oligomeric states from 140 up to 400 kDa (Schmidt et al., 2016). Thus, it remains unclear which oligomeric form(s) of S1R exists *in vivo* and which state(s) is(are) functionally active.

Sigma-1 receptor oligomerization is disrupted by mutations in the GXXXG motif corresponding to amino acid residues 87–91 (Gromek et al., 2014; Ortega-Roldan et al., 2015). The GXXXG motif was previously through to reside in the second transmembrane domain and mediate subunit association via transmembrane alpha helices. However, the crystal structure suggests that it forms a beta-hairpin structure inside the oligomerization interface (Schmidt et al., 2018). The distance between Cα atoms of Gly88 in each protomer is about 6 Å (Schmidt et al., 2018). Thus, mutations of this residue can sterically interfere with subunit association. Gromek et al. (2014) demonstrated that mutations within the GXXXG motif cause a shift toward the monomeric state of S1R. Interestingly, this is associated with a significant decrease in ligand binding, suggesting that ligand binding affinity may depend on S1R oligomerization processes. While oligomeric forms of S1R have a demonstrated ability to bind ligands, S1R monomers may lose this property (Gromek et al., 2014). Mutations within the GXXXG motif also decrease S1R expression, which may indicate reduced stability of GXXXG mutants (Gromek et al., 2014).

The oligomerization interface was further characterized with crystallographic data, leading to identification of key amino acids involved in subunit interactions. The sequence of the oligomerization interface is highly conserved among species, confirming its physiological importance. The trimerization surface is formed largely by hydrophobic residues within the C-terminal cupin domain. For example, a Phe191 residue from each protomer forms inter-subunit contacts. There are the polar interactions between sidechains of Thr141, His54, and Glu55. The amino acids Trp81, Phe83, Met90, Ala92 and Leu111, His116, Arg119, Trp136, Ala161, Trp169, Asp188, Phe191, Ser192, Gln194, Asp195, and Thr198 are also engaged in formation of the trimerization interface (Schmidt et al., 2016).

Despite the detailed characterization of oligomerization interface and comprehensive studies of ligand-induced structural rearrangements, the significance of S1R oligomer-monomer transitions in the regulation of S1R functions remains unknown. To correlate structural rearrangements observed *in vitro* with physiological responses, it has been proposed that the physiological significance of S1R oligomerization may be linked to the protein–protein interactions of S1R with its partners (Gromek et al., 2014; Yano et al., 2018). For example, the monomeric form of S1R interacts with Nav1.5, acid-sensing channels and D1R (Carnally et al., 2010; Navarro et al., 2010; Balasuriya et al., 2012). Two groups identified a direct and agonist-dependent interaction between S1R and the dopamine transporter (DAT), resulting in attenuated DA efflux and calcium signals evoked by methamphetamine (Hong W.C. et al., 2017; Sambo et al., 2017). Hong J. et al. (2017) suggested that agonists induce dissociation of S1R multimers into monomers which in turn interact with DAT. Mutational analyses have shown that the interaction site is located in the transmembrane domain of S1R.

As mentioned above, initiation of S1R activity was also proposed to involve ligand-, calcium-, or ER stress-dependent dissociation of S1R from binding immunoglobulin protein (BiP), which is a chaperone located in the lumen of the ER (Hayashi and Su, 2007). Recently, Yano et al. (2018) used a novel bioluminescence resonance energy transfer (BRET) assay to study the ligand-mediated oligomerization of S1R. They revealed the distinct effects of agonists and antagonists on S1R homomerization, consistent with previous results (Hong W.C. et al., 2017). Interestingly, while the agonist pentazocine facilitated interaction of BiP and S1R, haloperidol induced the dissociation of S1R from BiP. Thus, S1R ligands may regulate the association between S1R and BiP through controlling S1R oligomerization and monomerization. This is likely also relevant to S1R interactions with its client proteins (Figure 3).

It would be interesting to know more clearly how S1R associates with various proteins located in the ER lumen, ER membrane, cytoplasm and plasma membrane and to resolve the conflicting models of S1R topology and orientation. Given the topology model proposed by Mavylutov et al. (2018), the bulk of S1R may face the ER lumen. This topology is consistent with the well-described interaction of S1R with BiP, but raises it questions about how S1R interacts with proteins in the cytosol with only a small cytosolic N-terminal tail. Perhaps S1R has two or more structural elements or configurations responsible for the binding of S1R to different proteins. The structural and biological mechanisms of such interactions remain to be fully elucidated.

## S1R as a Target for Treating Neurodegenerative Diseases

Many S1R agonists are anti-amnestic, synaptogenetic, and neuroprotective in conditions of neuronal stress (Antonini et al., 2009; Hindmarch and Hashimoto, 2010; Ruscher et al., 2011; Bolshakova et al., 2016). They also mitigate disease and symptoms in experimental models of ALS (Mancuso et al., 2012; Ono et al., 2014; Peviani et al., 2014; Ionescu et al., 2019), Alzheimer’s disease (AD) (Meunier and Hayashi, 2010; Fisher et al., 2016; Maurice and Goguadze, 2017; Hall et al., 2018; Goguadze et al., 2019; Ryskamp et al., 2019), Parkinson’s disease (PD) (Francardo et al., 2014; Francardo et al., 2019) Huntington’s disease (HD) (Squitieri et al., 2015; Geva et al., 2016; Bol’shakova et al., 2017; Garcia-Miralles et al., 2017; Ryskamp et al., 2017; Kusko et al., 2018) stroke (Allahtavakoli and Jarrott, 2011; Ruscher et al., 2011, 2012; Sato et al., 2014; Urfer et al., 2014) and traumatic brain injury (Dong et al., 2016). By contrast, S1R deficiency exacerbates progression of neurological disorders (Mavlyutov et al., 2011, 2013; Ha et al., 2012; Francardo et al., 2014; Miki et al., 2015; Maurice et al., 2018) as well as symptoms commonly associated with neurodegenerative diseases. For example, pharmacological inhibition of S1R leads to mushroom spine loss in hippocampal cultures (Ryskamp et al., 2019) and this could be related to memory impairments from the anti-psychotic drug and S1R antagonist haloperidol (K_D_ for S1R ∼3 nM) (Abdel-Salam et al., 2012). S1R knockout (KO) mice have several phenotypes resulting from neuronal dysfunction and late-onset neurodegeneration (Sabino et al., 2009; Ha et al., 2011; Sha et al., 2015). These data collectively highlight the innate, neuroprotective properties of S1R activity. The following sections summarize genetic associations of S1R mutations/polymorphisms with neurodegenerative diseases, examples of neuroprotection in respective disorders by S1R agonists, and possible mechanisms of action.

## S1R in Amyotrophic Lateral Sclerosis/Frontotemporal Dementia (ALS/FTD)

Amyotrophic lateral sclerosis is a fatal neurodegenerative disease featuring progressive weakness of skeletal muscles due to upper and/or lower motor neuron dysfunction and loss. Several recessive, loss-of-function mutations in S1R have been associated with ALS, distal hereditary motor neuropathy and/or FTD (Luty et al., 2010; Al-Saif et al., 2011; Kim et al., 2014; Li et al., 2015; Ullah et al., 2015; Gregianin et al., 2016; Horga et al., 2016; Watanabe et al., 2016). S1R is highly expressed in motor neurons (Gundlach et al., 1986; Mavlyutov et al., 2010), suggesting a possible cell autonomous mechanism of motor neuron degeneration in these patients. Although S1R KO mice do not develop an overt ALS phenotype (Langa et al., 2003), they have deficits in locomotion and motor performance (Mavlyutov et al., 2010) related to muscle weakness, axonal degeneration, and motor neuron loss (Bernard-Marissal et al., 2015). KO of S1R accelerates the onset and progression of ALS in the SOD1^G93A^ mouse model of ALS (Mavlyutov et al., 2013), whereas the S1R agonists PRE-084 and SA4503 are neuroprotective and extend survival of SOD1^G93A^ mice (Mancuso et al., 2012; Ono et al., 2014). PRE-084 is also protective in wobbler mice, which develop spontaneous motor neuron degeneration (Peviani et al., 2014). PRE-084 is a derivative of phencyclidine (PCP) with nanomolar affinity for S1R and negligible affinity for PCP receptors and GPCRs (Su et al., 1991). SA4503 has low nanomolar affinity for S1R, low micromolar affinity for S2R, and little affinity for 36 other receptors, ion channels and second messenger systems (Matsuno et al., 1996). Treatment of SOD1^G93A^ mice with the S1R agonist pridopidine improves axonal transport (e.g., of BDNF, GDNF, and mitochondria) and BDNF secretion while attenuating atrophy of neuromuscular junctions, muscle fibers and motor neurons (Ionescu et al., 2019) (pharmacological properties summarized in HD section). Pridopidine treatment reduces the prevalence of SOD1 aggregates in spinal cords of SOD1^G93A^ mice (Ionescu et al., 2019). S1R activity may additionally protect motor neurons by reducing their excitability through facilitation of potassium channel activity (Mavlyutov et al., 2015b). Motor neuron degeneration from the absence of S1R is associated with reduced contacts between mitochondria and ER, ER stress, calcium dysregulation (Bernard-Marissal et al., 2015) and this may help to explain pathology in ALS patients with mutations in S1R.

## Role of S1R in Parkinson’s Disease (PD)

Dopamine receptors play important roles in learning and memory, motivation and movement and S1R agonists modulate dopaminergic signaling through multiple mechanisms. This has primarily been studied in the context of psychostimulant research, but these results may be important for understanding regulation of dopamine neurotransmission and its dysregulation in HD and PD. S1R appears to differentially regulate dopamine D1 and D2 receptors, as S1R activation by cocaine inhibits D2R (Navarro et al., 2013) and prevents histamine H3 receptor-dependent inhibition of the dopamine D1 receptor, stimulating Gs, recruitment of β-arrestin and phosphorylation of ERK1/2 (Moreno et al., 2014). Although S1R activation does not affect basal dopamine neurotransmission, it attenuates methamphetamine-induced and DAT-dependent increases in firing of dopamine neurons (Sambo et al., 2017). It also interacts directly with the DAT and attenuates calcium signals evoked by methamphetamine (Sambo et al., 2017). As a result, S1R limits hyperactivity, motivated behavior and reinforcement from methamphetamine (Sambo et al., 2017).

Abnormalities in movement and cognition in PD result from degeneration of dopaminergic neurons projecting from the substantia nigra to the striatum. S1R is expressed in these neurons (Hong W.C. et al., 2017) and it can bidirectionally modulate NMDAR-dependent release of dopamine in striatal brain slice experiments (Gonzalez-Alvear and Werling, 1994). S1R may be decreased in striatal regions that are preferentially affected in PD (Mishina et al., 2005), which could contribute to neuropathology as indicated by studies with S1R KO mice. Similar to PD patients, S1R KO mice have age-related deficits in motor behavior and degeneration of dopaminergic neurons (Hong W.C. et al., 2017). This appears to be related to aggregation and phosphorylation of α-synuclein which may be driven by phosphorylation of eIF2α from ER stress and proteasomal dysfunction (Hong W.C. et al., 2017). Pharmacological inhibition of ER stress prevented oligomerization of α-synuclein, dopaminergic neuron loss and motor impairments in S1R KO mice (Hong W.C. et al., 2017).

Recent studies found that S1R agonists are protective in PD models. For example, chronic treatment with PRE-084 gradually improves PD-like motor deficits from unilateral intrastriatal 6-hydroxydopamine (6-OHDA) lesions (hemiparkinsonian model) when treatment onset was prompt (Francardo et al., 2014). This treatment suppressed neuroinflammation while increasing levels of neurotrophic factors, monoamines (e.g., dopamine and serotonin), dopaminergic innervation of the striatum, and nigral neuron survival (Francardo et al., 2014). Low dose pridopidine treatment (0.3 mg/kg) of unilaterally 6-OHDA-lesioned mice partially protected nigral dopaminergic cell bodies and increased dopaminergic fiber density in the motor striatum (Francardo et al., 2019). This was associated with a gradual restoration of forelimb use (cylinder test, stepping test) and prevention of rotational bias toward the ipsilateral side (Francardo et al., 2019). The delayed recovery of motor function corresponds roughly with the expected timeline of pridopidine-dependent dopaminergic axon sprouting (Francardo et al., 2019). Treatment efficacy was absent in S1R KO mice, which had reduced loss of dopaminergic neurons in the substantia nigra pars compacta, but a greater loss of dopaminergic fibers in the striatum compared to wild-type mice (Francardo et al., 2019). The increased vulnerability of S1R knockout mice to axonal degeneration in the nigrostriatal pathway could relate S1R’s ability to promote growth and repair of neurites (Francardo et al., 2019). The neuroresorative effects of pridopidine were associated with upregulation of neurotrophic factors (BDNF, GDNF, pERK1/2) and associated signaling in the striatum and substantia nigra as well as reduced microglial activation (Francardo et al., 2019).

## Role of S1R in Huntington’s Disease (HD)

Huntington’s disease (HD) patients suffer from psychiatric, motor and cognitive disturbances that gradually worsen, leading to dementia, cachexia and eventually death. HD is a dominantly inherited neurodegenerative disease resulting from a CAG trinucleotide repeat expansion in exon 1 of the *Huntingtin* gene (>35 CAG repeats), leading to expression of mutant Huntingtin (mHtt) protein with an elongated polyglutamine tract. mHtt is broadly expressed throughout the body, but striatal MSNs are preferentially vulnerable in HD. The most significant contributions of mHtt to HD pathology remains a matter of debate and intense investigation. The CAG expansion compromises normal functions of Htt and disrupts cellular functioning through gain of mHtt function mechanisms (Imarisio et al., 2008; Kim et al., 2009), with possible toxic contributions from repeat-associated non-AUG translation (Banez-Coronel et al., 2015). This results in oxidative damage, glial reactivity, altered intracellular signaling, metabolism and energy levels, impaired axonal transport, transcriptional dysregulation, aberrant calcium regulation associated with ER stress, synapse loss and excitotoxicity (Zhai et al., 2005; Mochel and Haller, 2011; Leitman et al., 2013; Ryskamp et al., 2017).

Initial studies on the potential role of S1R in HD pathology were carried out with cellular models of HD. Hyrskyluoto et al. (2013), found that expression of mHtt (N-terminal huntingtin fragment proteins with 120 polyQ repeats or full-length Htt protein with 75 repeats) downregulates S1R expression in neuronal PC6.3 cells. There were no differences in S1R expression in control cells expressing the N-terminal fragment of Htt with 18 polyQ repeats or wild-type Htt. Administration of the selective S1R agonist PRE-084 prevented mHtt-dependent downregulation of S1R, SOD1, SOD2, thioredoxin 2, and Bcl-XL in neuronal PC6.3 cells (Hyrskyluoto et al., 2013). However, S1R expression appears to be upregulated in the striatum of YAC128 HD mice at 12 months of age and in the striatum of patients with advanced HD, possibly as an effort to compensate for ER calcium dysregulation and stress (Ryskamp et al., 2017). PRE-084 also decreased caspase-3 cleavage and oxidative stress and upregulated calpastatin, NF-κB-p65 levels and NF-κB signaling in mHtt expressing PC6.3 cells, enhancing their viability (Hyrskyluoto et al., 2013). Hyrskyluoto et al. (2013) proposed that the neuroprotective properties of S1R activity involved modulation of the calpastatin/calpain system, increasing NF-κB signaling and thereby upregulating antioxidants and decreasing ROS levels.

Another study demonstrated that large neuronal nuclear inclusions were strongly positive for S1R in human brains affected by polyglutamine diseases and intranuclear inclusion body disease (Miki et al., 2014). Also, S1R immunostaining colocalized with most intranuclear mHtt aggregates in HeLa cells expressing the N-terminal fragment of mHtt. Downregulation of S1R with antisense RNA increased the amount of mHtt aggregates in both the cytoplasm and nucleus. This was reproduced by treatment with the proteasome inhibitor epoxomicin. Moreover, proteasome activity was significantly lower following knockdown of S1R.

## Pridopidine’s Mechanism of Action in the Treatment of HD

Clinical trials with pridopidine indicate that it has efficacy in treating motor symptoms of HD (Lundin et al., 2010; De Yebenes et al., 2011; Esmaeilzadeh et al., 2011; Huntington, 2013; Reilmann et al., 2019) and recent evidence suggests that the therapeutic effect of pridopidine involves S1R. Pridopidine was originally dubbed a “dopamine stabilizer” based on behavioral experiments showing that it can both decrease locomotion in hyperactive rodents (e.g., from D-amphetamine or MK-801) and increase locomotion in hypoactive rodents (e.g., animals that have habituated to their environment or co-treated with the VMAT inhibitor tetrabenazine) (Ponten et al., 2010; Waters et al., 2014; Sahlholm et al., 2015). Increased locomotion in hypoactive rodents may relate to pridopidine’s ability to increase dopamine and norepinephrine in the cortex and subcortical areas (Ponten et al., 2010), which may also explain increased firing in prefrontal pyramidal neurons (Gronier et al., 2013). Pridopidine’s ability to bidirectionally normalize activity levels may have particular utility in the treatment of HD in which patients develop hyperkinetic motor disturbances followed by hypoactivity later in disease. The mechanism of action was initially proposed to involve low-affinity/fast-off negative modulation of dopamine D2 receptors with a slight binding preference for the agonist binding site when the receptor is in the active, catalytic, high-affinity state (Nilsson et al., 2004; Rung et al., 2008; Waters et al., 2014), but the affinity of pridopidine for D2 receptors of dopamine is relatively low being in the micromolar range (Dyhring et al., 2010). Unlike classical D2 receptor antagonists, pridopidine does not induce hypoactivity or catalepsy (Waters et al., 2018). Pridopidine also has micomolar affinity for several additional GPCRs including adrenergic alpha 2A/C receptors, serotonergic 5HT1A and 5HT2A receptors, and histamine H3 receptors (Gronier et al., 2013) and these interactions may influence levels of extracellular monoamines and glutamatergic neurotransmission (Waters et al., 2014). The effects of pridopidine in behavioral assays are not fully blocked in D2R receptor knockout mice (Svensson et al., 2009), prompting further investigation into potential molecular targets of pridopidine. More recently, pridopidine was found to have a high affinity (kD = ∼80 nM) for S1R (Sahlholm et al., 2013) and it primarily binds S1R rather than D2 receptors *in vivo* at behaviorally relevant doses (Sahlholm et al., 2015), suggesting that S1R might mediate the therapeutic effects of pridopidine.

Recent studies show that activation of S1R by pridopidine might be disease-modifying in HD. Squitieri et al. (2015) found that pridopidine reduces motor symptoms of R6/2 mice, improving performance on the horizontal ladder task and open-field locomotor measurements when treatment was started presymptomatically (5–6 mg/kg via daily intraperitoneal injections) (Squitieri et al., 2015). Pridopidine also extends their lifespan (Squitieri et al., 2015). *In vivo* treatment also normalized striatal BDNF and DARPP32 levels (Squitieri et al., 2015; Geva et al., 2016; Garcia-Miralles et al., 2017; Kusko et al., 2018), while decreasing the size and amount of mHtt aggregates (Squitieri et al., 2015). 150 μM pridopidine reduced apoptosis and restored pERK1/2 levels in a mouse striatal knock-in cellular HD model (STHdh111/111) and these effects were blocked by the S1R antagonist NE-100 (Squitieri et al., 2015). Ryskamp et al. (2017) found that low nanomolar concentrations of pridopidine and the structurally similar S1R agonist (+)3-PPP are neuroprotective in another cellular model of HD (Ryskamp et al., 2017). Both compounds stabilized synaptic connections between cortical and striatal MSNs in primary corticostriatal co-cultures prepared from from YAC128 HD mouse pups. Deletion of S1R with Cas9 prevented pridopidine and 3-PPP from rescuing dendritic spine loss in HD MSNs. Interestingly, S1R deletion also resulted in significant spine loss in WT MSNs. This observation indicated an important role for S1R in maintaining MSN spine stability. A synaptoprotective action of pridopidine was further supported by a series of Ca^2+^ imaging experiments. Previous studies demonstrated that abnormal Ca^2+^ signaling in post-synaptic spines is responsible for their destabilization in HD MSNs (Wu et al., 2016, 2018). Decreased ER Ca^2+^ levels due to mHtt-induced InsP_3_R1 hyperactivity (Tang et al., 2003, 2009) increases neuronal store-operated calcium entry (nSOC) in HD MSNs to synaptotoxic levels (Wu et al., 2011, 2016, 2018). Pridopidine treatment of corticostriatal co-cultures from YAC128 mice prevented InsP_3_R1 hyperactivity, restored ER Ca^2+^ levels, and decreased nSOC in HD MSNs (Ryskamp et al., 2017). Deletion of S1R WT MSNs resulted in depletion of ER Ca^2+^ content, suggesting that it might stabilize MSN spines through homeostatic control of ER Ca^2+^ levels (Ryskamp et al., 2017). Deletion of S1R in HD MSNs prevented the normalization of ER Ca^2+^ by pridopidine (Ryskamp et al., 2017). The selective S1R agonist PRE-084 also prevents dendritic spine loss in HD MSNs and this rescue is blunted by the S1R antagonist NE-100 (Bol’shakova et al., 2017). These findings suggest that in addition to the ability of pridopidine to mitigate motor symptoms of HD, it may also foster synaptic and neuronal viability via activation of S1R.

Consistent with this, Eddings et al. (2019) found that pridopidine and 3-PPP protects mouse primary striatal and cortical neurons from expression of mHtt (22 vs. 58 CAG repeats with the first 586 amino acids of Htt), as measured by imaging nuclear condensation in apoptotic cells and neuronal morphology. Pridopidine also rescued HD patient-derived induced pluripotent stem cells (Eddings et al., 2019). The S1R antagonist NE-100 or genetic ablation of S1R blocked the neuroprotective effects (Eddings et al., 2019). Although BDNF was also protective and is upregulated by S1R stimulation, blockade of BDNF signaling with the TrkB receptor antagonist ANA-12 did not impede the neuroprotective effects of pridopidine (Eddings et al., 2019). However, ANA-12, like NE-100, suppressed pridopidine’s ability to prevent mitochondrial depolarization from mHtt, as measured using tetramethyl rhodamine methyl ester (TMRM) (Eddings et al., 2019). These data indicate that S1R activation by pridopidine or 3-PPP is neuroprotective, but neuroprotection is not entirely mediated by BDNF signaling.

Pridopidine activates neuroprotective pathways that are compromised in HD (e.g., BDNF and AKT pathways), improving behavioral and transcriptional deficits in mouse models of HD (Geva et al., 2016; Garcia-Miralles et al., 2017; Kusko et al., 2018). Consistent with its ability to promote neuronal plasticity and survival, pridopidine upregulates expression BDNF, dopamine D1 receptor, AKT/PI3K and glucocorticoid pathway components and stimulates BDNF secretion in an S1R-dependent fashion (Geva et al., 2016; Kusko et al., 2018). Microarray and qPCR studies showed that pridopidine upregulates several genes downstream of BDNF including EGR1, EGR2, KLF5, CDKN1A, Homer1a, and Arc (Geva et al., 2016; Kusko et al., 2018). BDNF overexpression is sufficient to rescue many phenotypic characteristics of YAC128 HD mice (e.g., motor performance, cognitive deficits, synaptic density) (Xie et al., 2010), further suggesting that BDNF signaling could be an important contributor to neuroprotection following S1R activation. The idea that some of the beneficial effects of pridopidine in HD models can be mediated through BDNF signaling was supported by recent experimental evidence from Smith-Dijak et al. (2019). Synaptic scaling was suppressed in YAC128 cultures, as determined by recording the amplitude and frequency of mEPSCs after blockade of activity-dependent neurotransmission with TTX. Synaptic scaling was restored in YAC128 neurons by pharmacological activation of S1R with pridopidine or 3-PPP through BDNF-TrkB signaling (Smith-Dijak et al., 2019). Given that AKT is a potent pro-survival kinase, its upregulation may help promote neuronal resilience by phosphorylating apoptotic proteins (e.g., BAD and GSK3) and forkhead family transcription factors (e.g., FOXOs) (Geva et al., 2016; Kusko et al., 2018). Also, the calcium regulating genes calbindin and Homer1a are downregulated in the striatum of Q175 and YAC128 HD mice and they are both upregulated by pridopidine treatment (Ryskamp et al., 2017). These results indicate that when activated by pridopidine S1R acts on several transcriptional networks to foster neuronal function and survival in HD models.

A recent study demonstrated that pridopidine improves motor performance in YAC128 HD mice as well as anxiety- and depressive-like phenotypes, but it was unable to prevent striatal and corpus callosum atrophy (Garcia-Miralles et al., 2017), indicating that S1R agonism is insufficient to completely mitigate HD neuropathology and complementary treatment strategies should be considered. While pridopidine might be insufficient to completely prevent HD progression, when taken together the data on its effects in HD models and in HD patients shows that it can mitigate symptoms and is likely has the capacity to modify disease.

## Role of S1R in Alzheimer’s Disease (AD)

Alzheimer’s disease (AD) is the most pervasive cause of dementia in elderly people and it involves progressive impairment of memory and other cognitive faculties from damage to the hippocampus and other parts of the brain. Age is the main risk factor for the sporadic form of the disease. Early onset of AD is characterized by the development of the disease before the age of 65 and most of these cases result from autosomal dominant inherited mutations in amyloid precursor protein (APP), presenilin-1 (PSEN1) or presenilin-2 protein (PSEN2). Autosomal dominant inheritance accounts for about 1% of all cases of AD. When APP is cleaved by β- and γ-secretases, Aβ is formed with a length of 39 to 42 amino acid residues (Hardy, 2009). Presenilins are part of the γ-secretase protease complex and are key catalytic subunits. In AD mutations in the *APP* and *PSEN1*, *PSEN2* genes promote the formation of an extracellular fragment of Aβ with a length of 42 amino acid residues (Aβ_42_), the accumulation of which contributes to the formation of amyloid oligomers. Recently, it has been demonstrated that Aβ is generated intracellularly at the MAM domain and may influence functioning of the ER, mitochondria, and MAM (Schreiner et al., 2015). Given this finding and the importance of S1R at MAM domains (Hayashi and Su, 2007; Watanabe et al., 2016), it is perhaps not surprising that common S1R polymorphisms influence risk of developing AD (Uchida et al., 2005; Maruszak et al., 2007; Fehér et al., 2012). In fact, certain genetic combinations of S1R and apolipoprotein E (APOE) genotypes synergistically increase the risk of AD (Huang et al., 2011).

Several S1R agonists have anti-amnestic properties, overcoming learning and memory impairments from amyloid-β toxicity or scopolamine (Maurice and Goguadze, 2017). S1R agonists promote neurogenesis in the hippocampus (Moriguchi et al., 2013) and they may mitigate memory impairment because they can stabilize mature, mushroom spines (Ryskamp et al., 2019), which serve as sites of robust synaptic connections encoding lasting information (Bourne and Harris, 2007; Hayashi-Takagi et al., 2015). Mushroom spine loss may underlie memory defects in models of AD, as hippocampal neuron mushroom spines are lost *in vitro* and *in vivo* in both presenilin-1-M146V knock-in (PS1-KI) and APP knockin (APP-KI) models of AD (Sun et al., 2014; Zhang et al., 2015). As postmortem and *in vivo* brain imaging studies have demonstrated a reduced density of S1R in the brains of patients with AD (Jansen et al., 1993; Mishina et al., 2008) and S1R knockdown destabilizes mushroom spines (Tsai et al., 2009; Fisher et al., 2016; Ryskamp et al., 2019), downregulation of S1R may contribute to AD pathology. Consistent with this, knockout of S1R in APP_Swe_ AD mice increases oxidative stress within the hippocampus and exacerbates memory impairments (Maurice and Goguadze, 2017; Maurice et al., 2018). The novel positive S1R modulator (±)-2-(3-chlorophenyl)-3,3,5,5-tetramethyl-2-oxo-oxazaphosphinane (OZP002) (Gundlach et al., 1986; Maurice et al., 2006a; Tsai et al., 2009) was also neuroprotective in pharmacological and genetic models of AD. It potentiated the antidepressant effect of the S1R agonist igmesine and prevented scopolamine-induced learning deficits in the Y maze test and passive avoidance test. Its effect was blocked by NE-100 or in S1R knockout mice (Maurice et al., 2019).

Treatment of hippocampal cultures with Aβ_42_ oligomers induces loss of mushroom spines (Popugaeva et al., 2015; Zhang et al., 2015) and Aβ_42_ accumulation in hippocampal cultures prepared from APP knock-in mice also causes mushroom spine loss (Zhang et al., 2015). This is also observed *in vivo* (Zhang et al., 2015). Pridopidine and 3-PPP prevent mushroom spine loss from both of these sources of Aβ toxicity in hippocampal neuronal cultures (Ryskamp et al., 2019). Pridopidine treatment normalized synaptic functioning, preventing LTP deficits caused by Aβ_42_ oligomers (Ryskamp et al., 2019). Pridopidine and 3-PPP also prevented mushroom spine loss in hippocampal cultures prepared from PS1-KI mice (Ryskamp et al., 2019) that model familial AD (Guo et al., 1999). Importantly, oral treatment with pridopidine rescued mushroom spines *in vivo* in PS1-KI mice (Ryskamp et al., 2019), suggesting this as a viable treatment strategy for memory deficits in familial AD.

AF710B also stabilized mushroom spines *in vitro* in hippocampal cultures prepared from AD mice (PS1-KI and APP-KI models) (Fisher et al., 2016). AF710B was found to potently and selectively stimulate the M1 muscarinic acetylcholine receptor (M1R) and S1R (Fisher et al., 2016). AF710B binds to M1R outside of its orthosteric binding site, suggesting an allosteric mechanism of action (Fisher et al., 2016). This is supported by data showing that 0.1 nM AF710B enhances the affinity and potency of the M1R agonist carbachol (Fisher et al., 2016). The mechanism by which AF710B activates S1R is less clear, but the anti-amnestic properties of AF710B appear to require S1R, because the S1R antagonist NE-100 can suppress them (Fisher et al., 2016). No significant binding was observed with other targets in a screen involving 83 GPCRs, ion channels and transporters (Fisher et al., 2016). Treatment of 3xTg-AD mice with AF710B (10 μg/kg delivered by intraperitoneal injections daily for 2 months) reduced levels of BACE1, Aβ_1__–__42_, plaques, p25/CDK5, GSK-3β activity, Tau phosphorylation and memory deficits in the Morris water-maze (Fisher et al., 2016). It was previously known that M1R activation improves cognition and reduces AD-like pathology in animal models (Caccamo et al., 2006; Medeiros et al., 2011), but the combined activity of AF710B at both M1R and S1R might make it particularly therapeutic. Indeed, *in vivo* treatment of McGill-R-Thy1-APP transgenic rats also reduced amyloid burden and inflammation while enhancing synaptogenesis and cognition (Fisher et al., 2016). Another mixed muscarinic/σ1R agonist, ANAVEX2-73, was able to mitigate Aβ_25__–__35_-induced tau phosphorylation and Aβ_1__–__42_ seeding in mice (Lahmy et al., 2013) and may help to preserve cognition in preliminary clinical trials clinical studies with AD patients (Macfarlane et al., 2016).

In addition to S1R agonists, positive allosteric modulators that do not compete with the (+)-pentazocine binding site might have therapeutic value. For example, SKF-83959 shows promise in the 6-OHDA-induced rat model of Parkinson’s disease (Zhang et al., 2005, 2007; Guo et al., 2013). Also, OZP002 attenuated learning deficits from scopolamine, ICV injection of amyloid Aβ_25__–__35_, or the APP_Swe_ transgene and protected against neurotoxicity associated with ICV injection of amyloid Aβ_25__–__35_ (Vavers et al., 2019). Several selective allosteric modulators for S1R have been discovered (methylphenylpiracetam and SOMCL-668) (see Vavers et al., 2019 for a review) and it will be interesting to see whether they are efficacious in models of neurodegenerative diseases. Hinting at potential utility, SOMCL-668 enhanced (+)-SKF-10047-stimulated neurite growth and BDNF production in an S1R-dependent manner (Wang et al., 2016). Although both direct agonists and positive allosteric modulators may have therapeutic promise, it is unclear which are better candidates for clinical trials.

Recent data suggests that S1R functionally interacts with presenilin 1 (PS1) and presenilin 2 (PS2), which are implicated in AD. Although cleaved PS1 is the catalytic subunit in the γ-secretase complex, the holoprotein version of PS1 functions as a passive leak channel in the ER membrane (Tu et al., 2006; Nelson et al., 2007; Zhang et al., 2010). Similarly, PS2 forms a calcium leak channel in the ER (Tu et al., 2006; Nelson et al., 2007; Zhang et al., 2010). Many familial AD-causing mutations in either PS1 or PS2 disrupt tonic Ca^2+^ release from the ER via PS1 and PS2 leak channels, increasing the concentration of calcium in the ER (Tu et al., 2006; Zhang et al., 2010). Pridopidine promotes ER calcium homeostasis by decreasing luminal calcium levels in cultured hippocampal neurons from WT, PS1-KI and conditional presenilin double-knockout mice (PS1^flox/flox^/PS2^–/–^) infected with lenti-NLS-GFP as well as in neurons expression Cas9 and gRNA targeting the PS1 gene (Ryskamp et al., 2019). However, this effect is lost in PS1^flox/flox^/PS2^–/–^ hippocampal neurons infected with lenti-NLS-GFP-Cre (Ryskamp et al., 2019). Knockout of PS1, PS2, or PS1/2 causes mushroom spine loss in hippocampal neurons (Ryskamp et al., 2019). Consistent with functional data, pridopidine can compensate for this and restore mushroom spine integrity in presenilin 1 or presenilin 2 knockout neurons, but not in PS1/2 double knockout neurons (Ryskamp et al., 2019). Consistent with the spine loss phenotype in PS1 and/or PS2 KO neurons, inactivation of PS1 in the mouse forebrain causes mild cognitive deficits, whereas inactivation of both PS1 and PS2 severely impacts cognition and synaptic plasticity, leading to neurodegeneration (Sun et al., 2005). PS1 and PS2 both contribute to resting ER calcium homeostasis and form a redundant ER calcium leak pathway (Tu et al., 2006; Nelson et al., 2007) that is required for pridopidine to decrease the concentration of ER calcium and stabilize mushroom spines (Ryskamp et al., 2019). The reason for this is unclear, but S1R agonists might decrease ER calcium in hippocampal neurons by enhancing ER calcium leakage either by modulating leak activity of PS channels or increasing the prevalence of PS holoprotein in the ER membrane. The later possibility is intriguing because S1R might impede catalytic cleavage of PS1, reducing its incorporation into the γ-secretase complex. This could underlie the ability of S1R agonists to reduce Aβ_42_ accumulation and aggregation (Fisher et al., 2016). However, more work is needed to characterize how S1R activity normalizes ER calcium homeostasis and mushroom spine prevalence while mitigating other hallmarks of AD neuropathology.

When ER calcium levels are chronically elevated from mutations in presenilin 1, suppression of neuronal store-operated calcium entry (nSOC) leads to destabilization of hippocampal mushroom spines (Sun et al., 2014; Zhang et al., 2016). Upregulating nSOC either pharmacologically or via overexpression of STIM2 or EB3 prevents PS1-KI mushroom spine loss (Sun et al., 2014; Zhang et al., 2016; Pchitskaya et al., 2017). Consistent with pridopidine’s ability to decrease ER calcium levels in hippocampal neurons, it increased nSOC in the spines of cultured PS1-KI neurons and activity of the nSOC pathway was required for the rescue of PS1-KI mushroom spines (Ryskamp et al., 2019). This suggests that pridopidine rescues PS1-KI mushroom spines through decreasing ER calcium levels and thereby stimulating nSOC pathway activity.

Highlighting the importance of the client protein milieu in determining the effects of S1R activity, S1R suppresses store-operated calcium entry (SOC) in other cell types outside of the hippocampus. In non-neuronal cells, treatment with S1R agonists or overexpression of S1R or suppresses SOC (Brailoiu et al., 2016; Srivats et al., 2016). This may involve S1R binding to STIM1 and disrupting the interaction of STIM1 and Orai1 (Srivats et al., 2016). Additionally, in MSNs from YAC128 mice that model Huntington’s disease pridopidine decreases supranormal nSOC, which is synaptotoxic to MSNs (Ryskamp et al., 2017). This contrasts with data involving hippocampal neurons in which pridopidine decreased ER calcium levels and enhanced nSOC (Ryskamp et al., 2019). This divergence indicates that the effect of S1R in a given cell type depends on the availability of S1R interaction partners. For instance, InsP_3_R1 constitutes the primary ER calcium release pathway in MSNs (Wu et al., 2016), whereas presenilins preferentially mediate leakage of ER calcium in hippocampal neurons (Tu et al., 2006; Nelson et al., 2007; Zhang et al., 2010). Additionally, although S1R suppresses STIM1-dependent SOC in HEK293 and CHO cells (Srivats et al., 2016), STIM2 is the predominant regulator of nSOC in hippocampal neurons (Sun et al., 2014). S1R might not bind and sequester STIM2 the way it does with STIM1 or if S1R does bind STIM2, it might enhance or minimally effect STIM2-gated nSOC. Thus, regulation of synaptic plasticity by S1R is likely to be multifaceted and highly dependent on the cellular context.

## Conclusion

Sigma-1 receptor is incredibly versatile in its ability to foster neuronal homeostasis in the context of several neurodegenerative disorders. Several S1R agonists are FDA-approved (Ishikawa and Hashimoto, 2009), such as fluvoxamine (Nishimura et al., 2008) and donepezil (Maurice et al., 2006b) and they may be repurposed for the treatment of several neurodegenerative diseases. Additional S1R agonists such as pridopidine have shown promising results in preclinical studies and in clinical trials.

## Author Contributions

All authors listed have made a substantial, direct and intellectual contribution to the work, and approved it for publication.

## Conflict of Interest Statement

The authors declare that the research was conducted in the absence of any commercial or financial relationships that could be construed as a potential conflict of interest.
